# Nurses’ understanding of the necessity of core items recommended by the latest Utstein resuscitation registry template for in-hospital cardiac arrest: A cross-sectional study

**DOI:** 10.1016/j.resplu.2024.100757

**Published:** 2024-09-03

**Authors:** Chika Nishiyama, Sayaka Takenouchi, Yoshinori Okuno, Youhei Kakuda, Shigeru Ohtsuru

**Affiliations:** a Department of Critical Care Nursing, Kyoto University Graduate School of Human Health Science, Kyoto, Japan; b Department of Nursing Ethics, Kyoto University Graduate School of Human Health Science, Kyoto, Japan; c Department of Primary Care & Emergency Medicine, Graduate School of Medicine, Kyoto University, Kyoto, Japan

**Keywords:** In-hospital cardiac arrest, Utstein-style, Documentation, Nurse

## Abstract

**Background:**

It is essential for nurses, who are more likely to be first responders to cardiac arrest patients in hospitals, to understand the items that should be recorded when a cardiac arrest occurs to record the event accurately. We aimed to assess Japanese nurses’ understanding of the necessity of recording core items, as defined in the Utstein-style reporting template.

**Methods:**

We conducted a cross-sectional study using an anonymous, self-administered online questionnaire survey at Kyoto University Hospital. In addition to nurses’ understanding of the necessity of recording Utstein core items, we collected data on years of experience as a nurse, experiences of encountering in-hospital cardiac arrest (IHCA), and understanding and confidence in performing basic life support.

**Results:**

Of 1,202 eligible nurses, 492 participated, among whom 5.3% were aware of the Utstein-style reporting template. None of the items were considered “necessary” by all respondents. A documentation form listing the items to be recorded was requested by 86% of the respondents, and 82% reported having difficulties due to a lack of opportunities to learn how to write resuscitation documentation.

**Conclusion:**

We found that nurses lacked an understanding of the Utstein-style reporting template, which is critical for effective management and reporting of IHCA. Detailed and accurate documentation is crucial for improving outcomes in patients with IHCA. Effective education for nurses and development of a recording system are challenges that must be addressed in the future.

## Introduction

In-hospital cardiac arrest (IHCA) is an unexpected event in hospitalized patients. The survival rate of IHCA has improved over the past 20 years.^1^, ^2^ The incidence of IHCA is estimated to be 1–10 per 1,000 hospitalized patients, with a 30-days survival rate of approximately 25%.^3^, ^4^, ^5^, ^6^ However, the frequency of occurrence and survival rates vary widely across registries.^7^ This variation is attributed to differences in the following factors: data collection and reporting methods related to IHCA, cultural attitudes toward end-of-life care in various countries and regions, and implementation of “do not resuscitate” orders. Few randomized controlled trials have targeted IHCA, and evidence from patients with out-of-hospital cardiac arrest is often extrapolated or based on expert opinions. Resuscitation guidelines for IHCA have changed little over the past 5–10 years.^8^

The collection of complete and accurate IHCA records is essential for developing resuscitation guidelines and for continuous process improvement activities. The Utstein template enables uniform reporting of cardiac arrest data and defines IHCA as published in 1997.^9^ The International Liaison Committee on Resuscitation (ILCOR) significantly revised the items and their definitions to ensure accurate data collection. In September 2019, it released a revised version of the internationally standardized Utstein style for IHCA.^10^

Nurses have more consistent direct contact with patients than any other healthcare professional. This is not an exception during IHCA, where nurses are likely to be the first responders, and whether they can quickly notice abnormalities and start Basic Life Support (BLS) promptly contributes significantly to patient survival. Since cardiac arrest can occur anywhere and at any time, the Japan Resuscitation Council Guidelines 2020 emphasize that all medical personnel should be equipped with BLS skills.^11^ Recording resuscitation efforts and verifying the resuscitation process are simultaneously important. The ILCOR recently released a strategy statement, “Ten steps toward improving in-hospital cardiac arrest quality of care and outcomes”, which outlines 10 steps to improve care in IHCA.^12^ They emphasized the importance of collecting data to measure and enhance resuscitation processes and outcomes, noting that data collection relies on the accuracy of documentation by frontline professionals. Furthermore, they stated that each institution should conduct a thorough needs assessment of the current situation related to the IHCA before effectively implementing the Ten Steps. During emergencies, nurses are often responsible for the documentation of resuscitation. High-quality documentation is considered unattainable unless those responsible for recording understand what should be recorded and the significance of the items. However, to the best of our knowledge, no studies have investigated nurses' awareness of the latest Utstein-style reporting template. Therefore, we aimed to assess Japanese nurses' understanding of the necessity of recording core items for IHCA, based on the latest Utstein Resuscitation Registry Template for IHCA (Utstein-style reporting template).

## Methods

### Study design and population

We conducted a cross-sectional study using an anonymous, self-administered online questionnaire survey between February 14 and March 31, 2023. We included nurses, regardless of department, working at a national university hospital in Japan but excluded the director, four vice directors, and those on parental, maternity, or sick leave during the study period.

### Study setting

Kyoto University Hospital is a national university hospital with 1,141 beds (as of April 2023) and approximately 21,190 admissions/year.^13^ For cardiac arrests, immediate care is provided by ward staff, and the cardiac arrest team is notified via an emergency call system that includes the Department of Primary Care and Emergency Medicine physicians.

Nurses usually record interventions made during an emergency on a form permanently placed on the emergency cart and do not follow the Utstein-style reporting template. After the event, these records are entered into the medical record. Additionally, the electronic medical record system does not adhere to the Utstein-style reporting template, and there is no designated form. Consequently, nurses and doctors record each IHCA event's information separately in an open-ended format. Furthermore, no system is in place to register IHCA cases using an Utstein-style reporting template.

### Questionnaire and data collection

The original questionnaire was composed of 18 questions on the following two major topics: (1) nurses' characteristics, such as years since obtaining a nursing license, experience encountering IHCA and the number of encountered IHCA cases, certification for BLS or Advanced Life Support (ALS) instructor, understanding of BLS and ALS procedures, self-confidence in performing BLS and ALS, and experience learning how to write resuscitation documentation; and (2) nurses' perception of documentation of IHCA events, such as awareness of the existence of an internationally standardized documentation form for IHCA and awareness of the existence of the Utstein-style reporting.

The following items were included in the questionnaire to assess nurses' understanding of the necessity of recording core Utstein items. Of the 27 core items, 12 related to hospital information, the post-resuscitation process, and long-term outcomes were excluded, as nurses are unlikely to record these items during an emergency. Recognizing the critical nature of time during resuscitation, we included data on “Date/time of resuscitation team called,” “Date/time of chest compression,” and “Date/time of defibrillator shocks delivered” in the questionnaire for a total of 18 items (Supplementary questionnaire survey form). We presented participants with the above items and asked them about their perceived need to record each item. A 4-point rating scale was used to evaluate their perceived need to record each item, with a score of 1 for “Absolutely necessary,” 2 for “Necessary,” 3 for “Not very necessary,” and 4 for “Not necessary at all.”

Flyers detailing the purpose of the study, study period, and URL and QR codes for the survey form were distributed to all candidate nurses. Nurses who obtained the flyers voluntarily accessed and completed the survey during the study period.

### Statistical analysis

We aimed to assess nurses' understanding of the necessity of recording core items for IHCA events. To ensure the maximum validity of the results obtained, responses from all participants who agreed to participate in the survey were analyzed. As this was not a hypothesis-testing study, we carefully described the acquired data and identified trends in the results. Statistical tests were not performed. Data are summarized as numbers and percentages for categorical variables and median and interquartile range for numerical variables. All analyses were performed using the SPSS ver. 24.0 J (IBM Corp. Armonk, NY).

As all the Utstein items presented in this questionnaire are deemed essential for recording, respondents who answered, “Absolutely necessary” were classified as “necessary,” while all others were considered “unnecessary.” The respondents were surveyed on their understanding of and confidence in performing BLS and ALS, using a four-point scale. Those who selected “Understood well” for their understanding and “confident” for their ability to perform were defined as “Understanding and confident.”

### Ethical considerations

All procedures were conducted in accordance with the Declaration of Helsinki and Ethical Guidelines for Medical and Health Research Involving Human Subjects of Japan. Based on the Ethical Guidelines, participants who accessed the survey form read the study description displayed on the screen, and only those who agreed to participate completed the questionnaire. Voluntary participation and free withdrawal were also ensured. This study was approved by the Ethics Committee of Kyoto University Graduate School of Medicine (registration number R3827).

## Results

### Characteristics of participants

In total, 1,202 nurses were eligible. Of them, 492 (40.9%) responded to the questionnaire. The participant characteristics are presented in Table 1. Among the respondents, 53.7% had been licensed nurses for 11 or more years. Additionally, 13.4% were working in the critical care unit, 52.0% had encountered IHCA, and 44.7% had learned how to write resuscitation documentation. Only 26 (5.3%) responders were aware of the term “Utstein-style reporting template for IHCA”. Sixty-one individuals (12.4%) understood well and were confident about performing BLS, and 35 (7.1%) understood well and were confident about performing ALS (data not shown in Table 1).

**Table 1:** Participants' characteristics (n = 492).

	n	(%)
Years of experience as a nurse, year, median (IQR)	12	(4.3–21.0)
≤ 1 year	22	(4.5)
2–4 years	101	(20.5)
5–10 years	105	(21.3)
≥ 11 years	264	(53.7)
Working in critical care unit^a^	66	(13.4)
Experience of encountering IHCA	256	(52.0)
Number of encountering IHCA^b^		
One time	81	(32.1)
Two times	52	(20.3)
Three times	41	(16.1)
Four times	9	(3.5)
More than five times	73	(28.5)
Awareness of the Utstein-style reporting template for IHCA	26	(5.3)
Experience learning how to write resuscitation documentation	220	(44.7)
Opportunity to learn how to write documentation^c^		
Teaching from senior ward staff	109	(49.5)
In-hospital training	107	(48.6)
External seminars, conferences, BLS or ALS training course	53	(24.1)
Training at previous hospital	43	(19.5)
Self-learning	34	(15.5)
Pre-graduation educational program	17	(7.7)
Certification of BLS or ALS instructor	20	(4.1)
Understanding of BLS procedure		
Understand well	139	(28.3)
Mostly understand	297	(60.4)
Partially understand	50	(10.2)
Do not understand	6	(1.2)
Self-confidence in performing BLS		
Confident	66	(13.4)
Somewhat confident	156	(31.7)
Not very confident	209	(42.5)
Not confident	61	(12.4)
Understanding of ALS procedure		
Understand well	83	(16.9)
Mostly understand	247	(50.2)
Partially understand	147	(29.9)
Do not understand	15	(3.0)
Self-confidence in performing ALS		
Confident	35	(7.1)
Somewhat confident	111	(22.6)
Not very confident	235	(47.8)
Not confident	111	(22.6)

AED, automated external defibrillator; ALS, advanced life support; BLS, basic life support; CPR, cardiopulmonary resuscitation; IHCA, in-hospital cardiac arrest; IQR, interquartile range; ROSC, return of spontaneous circulation.

a Including ICU (intensive care unit), HCU (high care unit), CCU (cardiac care unit), ES-ICU (emergency & stroke intensive care unit), and ER (emergency room).

b Using data for those who encountered IHCA (n = 256).

c Using data for those who learned how to write documentation (n = 220). Multi-choice was allowed.

### Participants' needs and understanding of the necessity of the core items

Among the 18 items, none of the items were considered “necessary” by all respondents. Additionally, only eight of these items were deemed necessary by more than 90% of the respondents (Table 2). Of all the respondents, only 69 (14.0%) considered all the 18 items necessary, with only three responders (4.3%) being aware of the Utstein-style reporting template for IHCA. In contrast, among the 423 responders who did not consider all items necessary, only 23 responders (5.6%) were aware of the Utstein-style reporting template for IHCA (data not shown in Table 2).

**Table 2:** Participants' needs and understanding of the necessity of the core items (n = 492).

	n	(%)
Participants' needs		
A documentation form listing the items to be recorded	425	(86.4)
Opportunity of how to write resuscitation documentation		
Very troubled	136	(27.6)
Moderately troubled	269	(54.7)
Slightly troubled	73	(14.8)
No trouble	14	(2.8)
Necessity of the Utstein core items		
Patient		
Age	158	(32.1)
Sex	155	(31.5)
Pre-event data		
Subject type	275	(55.9)
Cardiac arrest process		
Date/time of event	457	(92.9)
Event location	337	(68.5)
Event witnessed	284	(57.7)
Resuscitation team called	426	(86.6)
Date/time of resuscitation team called	402	(81.7)
Monitored cardiac arrest	264	(53.7)
Chest compressions	457	(92.9)
Date/time of chest compression	464	(94.3)
Initial rhythm	395	(80.3)
AED used	446	(90.7)
Defibrillator shocks delivered	459	(93.3)
Date/time of defibrillator shocks delivered	451	(91.7)
Outcomes		
Date/time of CPR stopped	458	(93.1)
Reason CPR stopped	425	(86.4)
Any ROSC	467	(94.9)

AED, automated external defibrillator; CPR, cardiopulmonary resuscitation; ROSC, return of spontaneous circulation.

A documentation form listing the items to be recorded was requested by 86.4% of the respondents and 82.3% reported having difficulties (27.6%, very troubled; 54.7%, moderately troubled) due to a lack of opportunities to learn how to write resuscitation documentation.

### Factors that may influence the understanding of the necessity of the Utstein core items: years of experience as a nurse, encountering IHCA, and understanding and confidence in BLS

#### Years of experience as a nurse

Table 3 shows the understanding of the necessity of the Utstein core items based on years of nursing experience. Those who had worked longer as nurses were more likely to encounter IHCA or have awareness of the Utstein-style reporting template. Longer nursing experience did not necessarily correlate with a higher likelihood of the necessity of core items; the trend was almost the same regardless of years of experience.

**Table 3:** Participants' characteristics and understanding of the necessity of the core items based on their years of experience as a nurse (n = 492).

	≤ 1 year		2–4 years		5–10 years		≥ 11 years	
	n = 22		n = 101		n = 105		n = 264	
	n	(%)	n	(%)	n	(%)	n	(%)
Participants' characteristics								
Working in critical care unit^a^	3	(13.6)	6	(5.9)	19	(18.1)	38	(14.4)
Experience of encountering IHCA	3	(13.6)	31	(30.7)	44	(41.9)	178	(77.4)
Awareness of the Utstein-style reporting template for IHCA	0		2	(2.0)	2	(1.9)	22	(8.3)
Experience learning how to write resuscitation documentation	11	(50.0)	47	(46.5)	46	(43.8)	116	(43.9)
Certification of BLS or ALS instructor	0		4	(4.0)	6	(5.7)	10	(3.8)
Participants' needs								
A documentation form listing the items to be recorded	21	(95.5)	91	(90.1)	90	(85.7)	223	(84.5)
Opportunity of how to write resuscitation documentation								
Very troubled	11	(50.0)	35	(34.7)	27	(25.7)	63	(23.9)
Moderately troubled	11	(50.0)	59	(58.4)	56	(53.3)	143	(54.7)
Slightly troubled	0		6	(5.9)	20	(19.0)	47	(17.8)
No trouble	0		1	(1.0)	2	(1.9)	11	(4.2)
Necessity of the Utstein core items								
Patient								
Age	7	(31.8)	25	(34.7)	39	(37.1)	77	(29.2)
Sex	4	(18.2)	32	(31.7)	37	(35.2)	82	(31.1)
Pre-event data								
Subject type	13	(59.1)	71	(70.3)	62	(59.0)	129	(48.9)
Cardiac arrest process								
Date/time of event	21	(95.5)	95	(94.1)	97	(92.4)	244	(92.4)
Event location	16	(72.7)	67	(66.3)	70	(66.7)	184	(68.5)
Event witnessed	11	(50.0)	51	(50.5)	59	(56.2)	163	(61.7)
Resuscitation team called	18	(81.8)	91	(90.1)	95	(90.5)	222	(84.1)
Date/time of resuscitation team called	20	(90.9)	87	(86.1)	93	(88.6)	202	(76.5)
Monitored cardiac arrest	16	(72.7)	55	(54.5)	61	(58.1)	132	(50.0)
Chest compressions	21	(95.5)	96	(95.0)	103	(98.1)	237	(89.8)
Date/time of chest compression	21	(95.5)	98	(97.0)	101	(96.2)	244	(92.4)
Initial rhythm	18	(81.8)	81	(80.2)	92	(87.6)	204	(77.3)
AED used	20	(90.9)	96	(95.0)	101	(96.2)	229	(86.7)
Defibrillator shocks delivered	21	(95.5)	97	(96.0)	103	(98.1)	238	(90.2)
Date/time of defibrillator shocks delivered	19	(86.4)	95	(94.1)	100	(95.2)	237	(89.8)
Outcomes								
Date/time of CPR stopped	20	(90.9)	94	(93.1)	100	(95.2)	244	(92.4)
Reason CPR stopped	17	(77.3)	89	(88.1)	93	(88.6)	226	(85.6)
Any ROSC	21	(95.5)	97	(96.0)	102	(97.1)	247	(93.6)

AED, automated external defibrillator; ALS, advanced life support; BLS, basic life support; CPR, cardiopulmonary resuscitation; IHCA, in-hospital cardiac arrest; ROSC, return of spontaneous circulation.

a Including ICU (intensive care unit), HCU (high care unit), CCU (cardiac care unit), ES-ICU (emergency & stroke intensive care unit), and ER (emergency room).

#### Experience of encountering with IHCA

Those who encountered IHCA tended to have longer nursing experience and were more likely to have awareness of the Utstein-style reporting template compared to those who did not encounter IHCA. However, the understanding of the necessity of the core items was similar between those who had and had not encountered an IHCA (Supplementary Table 1).

#### Understanding and confidence in BLS

Those who understood and were confident in performing the BLS tended to have more nursing experience and were more likely to have experienced IHCA. However, the understanding of the necessity of core items was similar between the groups, except for the “Event witnessed” item (Supplementary Table 2).

## Discussion

We assessed nurses' understanding of the necessity of recording core items based on the latest Utstein template for IHCA. None of the 18 core items of the Utstein-style reporting template for IHCA were considered “necessary” by all responders. Additionally, only 14% of the respondents understood all the items as necessary, and only 5.3% of the respondents were aware of the Utstein-style reporting template. Further, over 80% of the respondents reported difficulties owing to a lack of opportunities to learn how to write resuscitation documentation.

Only 69 responders (14.0%) were aware of the Utstein-style reporting template, highlighting a lack of recognition among nurses working at a Japanese national university hospital. To the best of our knowledge, no study has investigated nurses' awareness of the Utstein-style reporting template. Therefore, we could not determine whether this percentage was low or high. However, this figure is lower than expected. Previous studies have highlighted the poor quality of resuscitation records,^14^ especially regarding the timing of events.^15^ Alarmingly, nurses, who are likely to identify cardiac arrest in patients and are responsible for resuscitation documentation, were not aware of the Utstein-style reporting template and do not understand what should be recorded. ILCOR suggests that the number of data items collected can be adjusted based on the organization's priorities and recommends that data collection be aligned with standardized definitions and items of the Utstein-style reporting template.^12^ Collecting data based on the Utstein-style reporting template allows for 1) consistent and comprehensive data collection, 2) visibility of the resuscitation process leading to quality improvement, and 3) objective evaluation of facility care through direct comparison with other facilities, regions, and countries using the Utstein-style reporting template.^10^ A standardized format allows high-quality data collection without missing patient outcomes or resuscitation process details required for post-event analysis. If the data entered by nurses is not properly recorded, standardized forms will not be utilized effectively. Thus, it is crucial for hospital directors and department leaders to accurately understand the necessity of using the Utstein-style reporting template. They should prepare standardized recording templates and establish a system that ensures that no data entry is missing. Nurses also indicated a preference for a documentation format that lists the items to be recorded rather than a free-form style. Therefore, in addition to promoting an understanding of the Utstein-style reporting template among nurses who are likely to identify cardiac arrest in patients and take initial action, it is important to create systems capable of collecting necessary data regardless of the nurses’ level of understanding of its necessity.

Even among the respondents who considered all the items “necessary,” only three were aware of the Utstein-style reporting template. As such, it is possible that most of the nurses did not respond “necessary” based on their knowledge of the Utstein-style reporting template, but rather out of experience or intuition. Since cardiac arrest can happen anytime and anywhere, all nurses should be familiar with the Utstein-style reporting template. In educating nurses on the Utstein-style reporting template, the team to be given priority should be well identified. Among those who had experience learning how to write resuscitation documentation, only 7.3% (16/220) were aware of the Utstein-style reporting template. Additionally, among those qualified to teach BLS or ALS, only 15% (3/20) understood, revealing a lack of awareness among instructors. Considering the finding that many people learn about resuscitation documentation from senior staff in the ward or through internal training, one strategy might be to initially target nurses involved in resuscitation education, including BLS and ALS instructors, to ensure that they acquire accurate knowledge of the Utstein-style reporting template and understand the core items.

The necessity of the mandatory items “age,” “gender,” and “patient type” was particularly unrecognized. As these items can be retrieved from post-event medical records, they may not have been recorded at the resuscitation site. This compels us to acknowledge that there is an issue with how we posed our questions. Nurses need to understand the necessity of documenting these items because potential victims of IHCA can vary, including outpatients, patients' families, visitors, and staff members.

Regardless of years of experience as a nurse, experience with IHCA, or understanding and confidence in performing BLS, awareness of the Utstein-style reporting template was less than 10% across all groups, and recognition of the necessity for core item recording showed similar trends. Longer experience as a nurse might increase exposure to the Utstein-style reporting template, but this does not necessarily correlate with higher recognition of the necessity of all core items. Given the low understanding and recognition of its necessity, promoting awareness of the Utstein-style reporting template is essential, regardless of nursing experience. Those who had experience encountering IHCA were more likely to be aware of the Utstein-style reporting template; however, the causal relationship between encountering IHCA and understanding the Utstein-style reporting template was unclear from this study design. Even among those who felt confident and performed the BLS, it was difficult to say that they understood the necessity of documenting the core items. Considering these findings, educating all nurses about Utstein-style reporting templates is vital, as cardiac arrest can occur anytime and anywhere. It may be beneficial to use the opportunities of regular BLS and ALS training, as well as mandatory hospital training sessions on medical safety, as occasions to educate nurses on the Utstein-style reporting template.

This study has several limitations. First, the results were obtained at a single hospital. Therefore, generalizing these findings to other hospitals may be inappropriate. In the absence of studies evaluating nurses' understanding of the latest Utstein-style reporting template, we believe it was appropriate, albeit conducted within a single hospital, to assess the current situation. Second, there might be an overestimation of the results because those interested in resuscitation, who have experienced IHCA, or who are involved in cardiac arrest education may have been more likely to respond, whereas those who could not may have been underrepresented in this survey. To increase the collection rate as much as possible, we informed the ward managers about the collection rate during the response period and asked them to encourage their staff members to respond. Third, the study focused on nurses most likely to encounter IHCA but did not assess awareness among other healthcare professionals. Given that cardiac arrest can occur anytime and anywhere, it is necessary to ensure that all staff have a foundational understanding of the Utstein-style reporting template for recording. Future studies should include other professions as well. Finally, this study did not investigate actual recording practices of nurses or patient outcomes. Considering that even those aware of the Utstein-style reporting template may not understand the core items, it is conceivable that there are deficiencies in actual documentation. We plan to assess the actual resuscitation documentation and then conduct interventional studies on recording training.

## Conclusion

Most nurses did not understand the Utstein-style reporting template. As accurate and comprehensive documentation is crucial for evaluating the factors contributing to IHCA, preventing cardiac arrest, and improving treatment/care, revisiting effective educational content and methods for nurses is an important issue to be addressed in the future.

## CRediT authorship contribution statement

**Chika Nishiyama:** Writing – review & editing, Writing – original draft, Visualization, Resources, Project administration, Methodology, Investigation, Funding acquisition, Formal analysis, Data curation, Conceptualization. **Sayaka Takenouchi:** Writing – review & editing, Writing – original draft, Methodology, Investigation, Conceptualization. **Yoshinori Okuno:** Writing – review & editing, Writing – original draft, Methodology, Investigation, Conceptualization. **Youhei Kakuda:** Writing – review & editing, Writing – original draft, Methodology, Investigation, Conceptualization. **Shigeru Ohtsuru:** Writing – review & editing, Supervision, Conceptualization.

## Declaration of competing interest

The authors declare that they have no known competing financial interests or personal relationships that could have appeared to influence the work reported in this paper.
