# Supplementary material for: Nurses’ understanding of the necessity of core items recommended by the latest Utstein resuscitation registry template for in-hospital cardiac arrest: A cross-sectional study
Source: Resusc Plus. 2024 Sep 3;20:100757. doi: 10.1016/j.resplu.2024.100757 (PMC11404068; doi:10.1016/j.resplu.2024.100757)
Supplement: Supplementary Data 1 [file mmc1.docx]

**Supplementary questionnaire survey form**

**Awareness of all nurses regarding in-hospital cardiac arrest records**

1. Have you experienced unexpected in-hospital cardiac arrest (e.g., sudden postoperative change, complications associated with an examination, asphyxiation, or unexpected cardiac arrest)?

Yes / No

2. If you selected "yes" for Question 1.

　　How many times have you encountered in-hospital cardiac arrest?

3. Lifesaving procedures required during in-hospital cardiac arrest include BLS (chest compressions, rescue breathing, and electrical shocks using an AED or defibrillator) and ALS (adding to BLS with airway interventions and adrenaline administered). Do you understand these procedures?

| Items | Understood well | Mostly understood | Not very well understood | Not understood (forgotten) |
| --- | --- | --- | --- | --- |
| BLS procedure | 1 | 2 | 3 | 4 |
| ALS procedure | 1 | 2 | 3 | 4 |

4. Do you feel confident in performing BLS and ALS techniques when encountering in-hospital cardiac arrest?

| Items | Confident | Somewhat confident | Not very confident | Not confident |
| --- | --- | --- | --- | --- |
| Performing BLS | 1 | 2 | 3 | 4 |
| Performing ALS | 1 | 2 | 3 | 4 |

5. Do you understand what information should be recorded when you encounter an in-hospital cardiac arrest?

Understood well / Mostly understood / Not very well understood /Not understood (forgotten)

6. Imagine that you were involved in resuscitation interventions for in-hospital cardiac arrest. Please select the number that applies to the need to record each of the following.

| Items | Example of description | Absolutely necessary | Necessary | Not very necessary | Not necessary at all |
| --- | --- | --- | --- | --- | --- |
| Age |  | 1 | 2 | 3 | 4 |
| Sex |  | 1 | 2 | 3 | 4 |
| Subject type | Outpatients, inpatients, staff, healthcare supply delivery personnel, etc. | 1 | 2 | 3 | 4 |
| Date and time of cardiac arrest |  | 1 | 2 | 3 | 4 |
| Location of cardiac arrest |  | 1 | 2 | 3 | 4 |
| Witnessed cardiac arrest | A cardiac arrest that is seen or heard by another person or is  monitored. | 1 | 2 | 3 | 4 |
| Resuscitation team called |  | 1 | 2 | 3 | 4 |
| Date/time of resuscitation team called |  | 1 | 2 | 3 | 4 |
| Monitored cardiac arrest | Monitoring already in place when need for chest compressions  and/or defibrillation was first recognized | 1 | 2 | 3 | 4 |
| Chest compressions |  | 1 | 2 | 3 | 4 |
| Date/time of chest compression |  | 1 | 2 | 3 | 4 |
| Initial rhythm | The first documented rhythm is the cardiac rhythm present  at the onset of cardiac arrest if monitored or when the monitor  or defibrillator is attached to the patient after the onset of chest  compressions. | 1 | 2 | 3 | 4 |
| AED used |  | 1 | 2 | 3 | 4 |
| Defibrillator shocks delivered |  | 1 | 2 | 3 | 4 |
| Date/time of defibrillator shocks delivered |  | 1 | 2 | 3 | 4 |
| Date and time CPR stopped |  | 1 | 2 | 3 | 4 |
| Reason CPR stopped | Death, DNAR | 1 | 2 | 3 | 4 |
| Any ROSC |  | 1 | 2 | 3 | 4 |

7. Are you aware that the items that should be recorded when in-hospital cardiac arrest occurs are internationally standardized?

Yes / No

8. Have you ever heard of the term "Utstein-style reporting template for in-hospital cardiac arrest "?

Yes / No

9. For documenting an in-hospital cardiac arrest, do you prefer a format with listed items that need to be recorded or a blank format that allows for free-form documentation?

A documentation form listing the items to be recorded / Free-form documentation

10. Have you ever learned about the necessity, significance, and content that should be documented during an in-hospital cardiac arrest?

Yes / No

11. Those who selected "yes" in Question 10

　　Where did you learn? Please select all that apply.

- - 1. Kyoto University Hospital
    2. Training at a previous hospital
    3. Pre-graduate education at universities and other institutions
    4. Teaching from senior ward staff
    5. External seminars, conferences, BLS or ALS training course
    6. Self-learning
    7. Other

12. Nurses can encounter an in-hospital cardiac arrest at any time. Please tell us the extent to which you find the following aspects challenging when responding to patients with in-hospital cardiac arrest:

| Items | Very troubled | Moderately troubled | Slightly troubled | No trouble |
| --- | --- | --- | --- | --- |
| Opportunities to learn BLS skills | 1 | 2 | 3 | 4 |
| Opportunities to learn ALS skills | 1 | 2 | 3 | 4 |
| Opportunity of how to write resuscitation documentation | 1 | 2 | 3 | 4 |
| Response to acute changes in patients with DNAR orders | 1 | 2 | 3 | 4 |
| Care for families of patients who have experienced cardiac arrest | 1 | 2 | 3 | 4 |
| Support for staff involved in in-hospital cardiac arrest | 1 | 2 | 3 | 4 |

13. How many years have you been a licensed nurse?

14. Please select your current working department.

15. Are you a certified BLS or ALS instructor such as AHA or ICLS?

　　　I have it. /I had it, but it had expired. / I did not have it.

16. Have you attended any BLS or ALS training sessions, excluding the BLS training you received when you joined the hospital?

Yes / No

17. Please tell us the clinical area in which you have worked the longest.

Cardiology / Pulmonology / Obstetrics / Emergency Medicine / ICU/CCU or other critical care

Other internal medicine / Other surgery / Home care, nursing facilities

18. Do you have any work experience in hospitals other than Kyoto University Hospital? Please answer regardless of the size, type, employment status, or department of the hospital.

Yes / No
